# Adequacy of nitisinone for the management of alkaptonuria

**DOI:** 10.1016/j.amsu.2022.104340

**Published:** 2022-08-07

**Authors:** Khawar Abbas, Jawad Basit, Mohammad Ebad ur Rehman

**Affiliations:** a Department of Surgery, Rawalpindi Medical University, Rawalpindi, Pakistan; b Department of Medicine, Rawalpindi Medical University, Rawalpindi, Pakistan

**Keywords:** Alkaptonuria, Nitisinone, Homogentisic acid, Ochronosis, Hypertyrosinemia, AKU, Alkaptonuria, HGA, Homogentisic acid

## Abstract

Alkaptonuria is a rare hereditary disease with a defective enzyme that results in increased homogentisic acid levels in the body. Homogentisic acid accumulates in multiple body parts and initializes tissue damage. Clinical manifestations such as pigmentation of the skin areas and joint destruction result in ochronosis. Nitisinone decreases serum and urinary homogentisic acid levels, improving morbidity by preventing and slowing the progression of alkaptonuria. Nitisinone-induced hypertyrosinemia causes keratopathy and mental ill effects, which can be managed by diet restriction and regular check-ups. A personalized approach is required for treatment by nitisinone. Low-dose oral nitisinone is associated with overall good results and a better safety profile.

## 1 Introduction

Alkaptonuria (AKU) is a rare hereditary metabolic disease with an autosomal recessive inheritance pattern. The cause is the defect in an enzyme homogentisic acid oxidase which involves the metabolism of homogentisic acid (HGA), an intermediate compound in the metabolism of tyrosine and phenylalanine. The characteristic findings are large joint arthritis, ochronosis, and homogentisic aciduria. Oxidation of increased HGA in urine turns it dark, which can be noticed in passing or stagnant urine. Though many patients don't see dark urine during their childhood, the disease comes into focus when they seek medical attention for resultant arthritis during their teens or adulthood [1].

HGA progressively accumulates in tissues which manifest in the form of pigmentation of the sclera, exposed parts of the skin, ear, etc. [1]. Cartilaginous tissue, mainly affected by the pathogenic pigmentation and cartilaginous structures in multiple body areas, becomes brittle and prone to fractures such as progressive arthritis of large synovial joints and intervertebral disc degeneration and subsequent kyphoscoliosis. All these clinical features are termed ochronosis. Nitisinone is the only effective drug that lowers HGA levels and prevents ochronosis [2].

The disease is rare, and less epidemiological data is available. The worldwide prevalence of AKU is 1 per 100,000 to 250,000. Alkaptonuria Severity Score Index (AKUSSI) is a scoring system based on clinical features that can objectively measure the degree of severity of the disease [3]. As far as our regional demographics are concerned, in a literature review from 1996 to 2016, eight patients were identified as having male predominance with positive family history in four cases. There was only an infant among all eight cases [4]. Furthermore, between 2014 and 2019, Biochemical Genetics Laboratory (BGL) at Aga Khan University Hospital reported twenty-one cases with a mean age of 19.4 ± 24.5 years and a male to female ratio of 2:1, of which eight were adults and twelve were below 2.5 years of age [5].

Nitisinone is an inhibitor of 4-Hydroxyphenylpyruvate dioxygenase and is used for treating hereditary tyrosinemia type 1. It is given orally in the dosage form of a capsule with a half-life of 54 h, 95% plasma protein binding capacity, is metabolized by CYP3A4, and excreted by the urine. It can be given to all ages, and there are no contraindications to its usage. Orphan drug status has been assigned to it. As nitisinone usage is associated with increased plasma tyrosine levels, a diet deficient in tyrosine and phenylalanine is recommended [6,7].

## 2 Nitisinone; a promising agent for alkaptonuria

AKU is being managed by supportive treatment both medically; pain killers, anti-inflammatory drugs, anti-oxidants i.e. ascorbic acid, and low tyrosine and phenylalanine diet as well as surgically; arthroplasty [8]. Investigation trials are going on to determine the adequacy of nitisinone [9]. A randomized controlled trial concluded that a 2 mg daily dosage of nitisinone effectively reduced plasma and urinary HGA levels to 95%, but the clinical parameters like the full hip range of motion and bone density were not significantly improved at this dose concentration. This poor result regarding clinical parameters may be due to a low dosage of nitisinone [10]. Similarly, a study conducted for four weeks proved the dose-response relationship as increasing nitisinone dosage concentration results in the decrease of 24-hr urinary HGA levels with the maximal effect obtained at 8 mg dose, which showed a 98.8% reduction in urinary HGA levels [5]. Another study found the same results at a 10 mg dose, which the European Medicines Agency has authorized to treat adult alkaptonuric patients [11].

In an observational trial, patients were monitored for three years to determine the effect of a 2 mg nitisinone once-daily dosage on disease progression. It was found that disease advancement declined after this period of treatment. Moreover, otic and ocular ochronosis was also seized, along with a decrease in the rate of change in the Alkaptonuria Severity Score Index (AKUSSI) score [12]. Another similar but large-scale study carried out at three different sites showed that 10 mg once daily dose of nitisinone specifically reversed the process of ochronosis in the ear with the arrest of the ochronotic process in the eye. There was a decline in Alkaptonuria Severity Score Index (cAKUSSI) score change among the treatment group of patients. Also, pain in the spine was reduced, and there was decreased incidence of tendon ruptures in this group of patients compared to the control group [13]. So, these studies showed that nitisinone is efficacious for treating AKU in adulthood. When nitisinone is used in childhood, it can appear as an effective preventive cure for complications related to AKU. A study by AKU Society, Cambridge, UK, is in the process of finding the age at which ochronosis most likely starts. This will clarify the best possible early age to start treatment with nitisinone and stop the disease before it appears [14].

## 3 Adverse effects of nitisinone

As every pharmaceutical agent has somewhat adverse effects, nitisinone is also not an exception in this case. One of the adverse effects is the increase in the plasma concentration of tyrosine. Hypertyrosinemia is, among others, problematic for the eyes and mental functioning. Tyrosine begins accumulating in aqueous humor and its level in aqueous humor exceeds plasma. The posterior corneal layer is leaky compared to the anterior epithelial layer, so tyrosine can easily enter the corneal stroma and cause keratopathy. Tyrosine-induced keratopathy may be asymptomatic, i.e. painless eye, or symptomatic, i.e. redness, pain, and photophobia, and sometimes hypertyrosinemia does not cause keratopathy at all. Diet restricted in tyrosine, and phenylalanine in the form of reduced protein intake and plasma tyrosine level measurements are recommended to prevent hypertyrosinemia. Moreover, regular slit-lamp examinations are necessary to timely notice any ocular change. If symptoms appear, nitisinone may be discontinued [15,16].

Plasma tyrosine is transported across the blood-brain barrier through a concentration gradient and enters the brain parenchyma and cerebrospinal fluid. It impairs DNA repair processes and elevates oxygen free radicals, which ultimately cause brain dysfunction [17]. It seemed in a retrospective study that higher levels of tyrosine affected cognitive abilities and brain functions related to schooling, such as memory, socializing, and learning skills in children [18]. Another study found that cognitive functions such as working memory deteriorated among older patients in a dose-dependent fashion upon tyrosine administration [19]. Contrary to this, an investigation inferred that rather than high tyrosine, low phenylalanine during the early days of life causes neurological retardation [20]. As for other adverse effects, diet restriction for tyrosine and phenylalanine and mental functions monitoring during nitisinone treatment is suggested to treat neuropathological changes [15].

## 4 Some ambiguities regarding treatment by nitisinone

Serum and urine HGA levels do not correlate significantly with the development of AKU complications in patients. There is variability in HGA levels and the clinical course of AKU among individuals. Therefore, presently it is unknown which reduced levels of HGA prevent ochronosis. Consequently, it cannot be said that a specific and fixed dosage of nitisinone will be useful for AKU patients [5]. Further interventional trials with a large sample size are required to develop a strategy to adjust the dosage of nitisinone according to individual needs keeping in mind the manner of the clinical course of disease among each patient.

As is already discussed, serum tyrosine levels rise in a dose-dependent fashion upon nitisinone therapy. So low dose of nitisinone will be beneficial as it will cause less rise in serum tyrosine levels, and consequently, there will be a low incidence of hypertyrosinemia-induced adverse effects. By increased nitisinone dosage, HGA levels can be reduced to a minimum, but tyrosine levels will rise on the other side. Overcoming this low dose of nitisinone can prove beneficial as it is also effective to reduce HGA levels by 90%, which is a better outcome [5,10,15].

As early age therapy with nitisinone proved beneficial in preventing ochronosis, this early age and long-term therapy may raise safety concerns for the patients, which need to be addressed in further studies. Combination therapy with anti-oxidants and devised protein restriction protocols should be considered with nitisinone dosage to control AKU outcomes efficiently [15].

Nitisinone therapy can only be given on an elective basis by prescribing the oral dose of the drug, as shown by some critical studies [9,13], and it cannot be administered in an emergency as it can only work with the progression and prevention of the disease if given for a longer duration.

## 5 Conclusion

In several investigations, nitisinone is proved to be beneficial in preventing the development of ochronosis in childhood and treating the complications of AKU if started in early adulthood. Adverse effects related to the said drug can be managed with regular follow-up and appropriate laboratory investigations. A proper treatment strategy can be devised for patients through further research. Low-dose nitisinone with combination therapy may be effective in treating AKU.

## Ethical approval

Not applicable.

## Sources of funding

No funding required for the study.

## Author contributions

Khawar Abbas: Study conception, write-up, critical review and approval of the final version.

Jawad Basit: Write-up, and approval of the final version.

Mohammad Ebad ur Rehman: Write-up, and approval of the final version.

## Registration of research studies

Name of the registry: Not applicable.

Unique Identifying number or registration ID: Not applicable.

Hyperlink to your specific registration (must be publicly accessible and will be checked): Not applicable.

## Guarantor

Khawar Abbas, Department of Surgery, Rawalpindi Medical University, Rawalpindi, Pakistan.

## Consent

Not required.

## Provenance and peer review

Not commissioned, externally peer reviewed.

## Declaration of competing interest

All authors declared no conflict of interest.
